# Supplementary material for: Patterns of Healthcare Resource Utilisation of Critical Care Survivors between 2006 and 2017 in Wales: A Population-Based Study
Source: J Clin Med. 2023 Jan 21;12(3):872. doi: 10.3390/jcm12030872 (PMC9917699; doi:10.3390/jcm12030872)

Supplemental material

Patterns of Healthcare Resource Utilisation of Critical Care Survivors between 2006 and 2017 in Wales: A Population-Based Study

Diagnostic plots

Figure S1: Factors associated with 1-year post-critical care HRU.

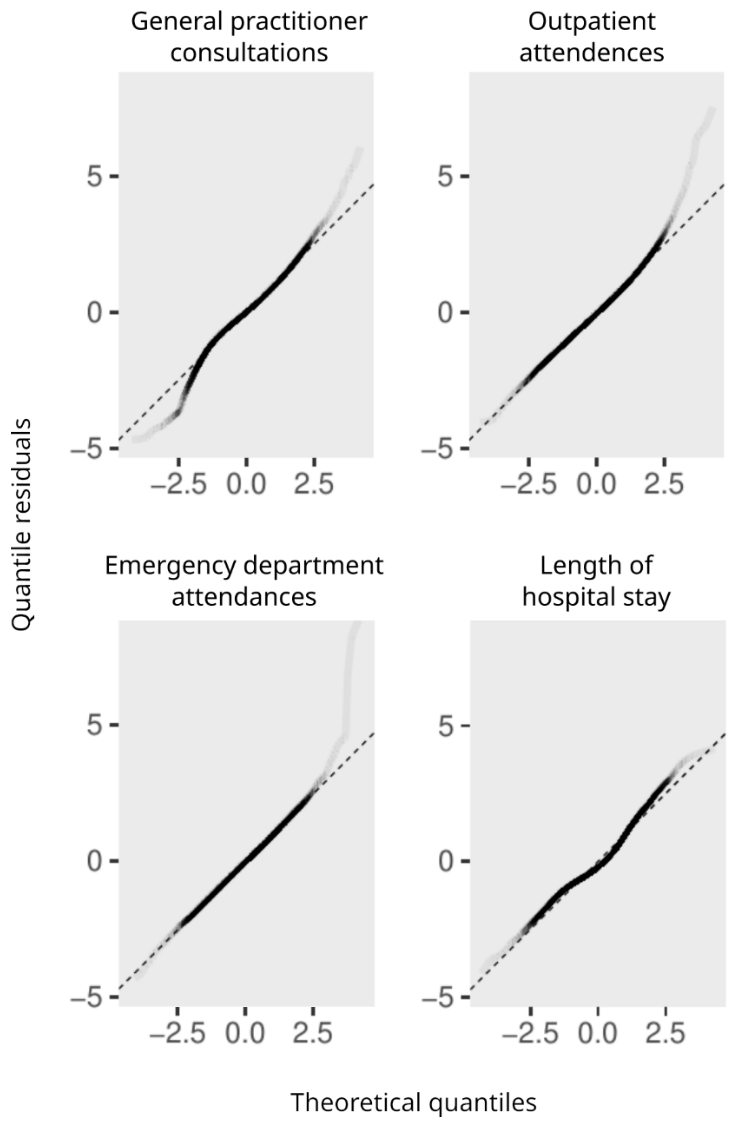

Figure S2: Predictors of change in health resource utilisation after critical care.

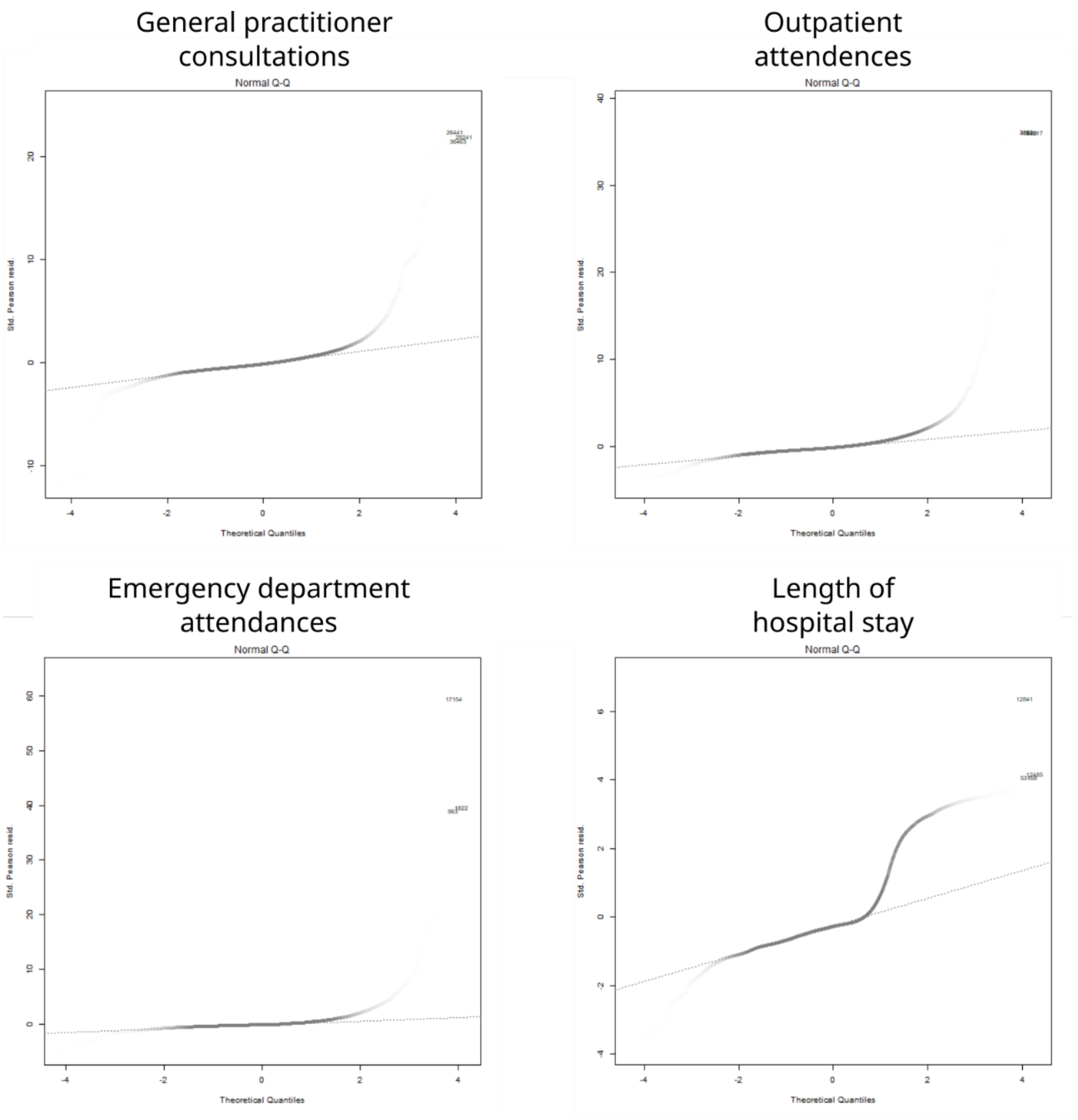

Figure S3: Association between critical care admission and health resource utilisation within 1 year of discharge from hospital.

(A) GP consulations

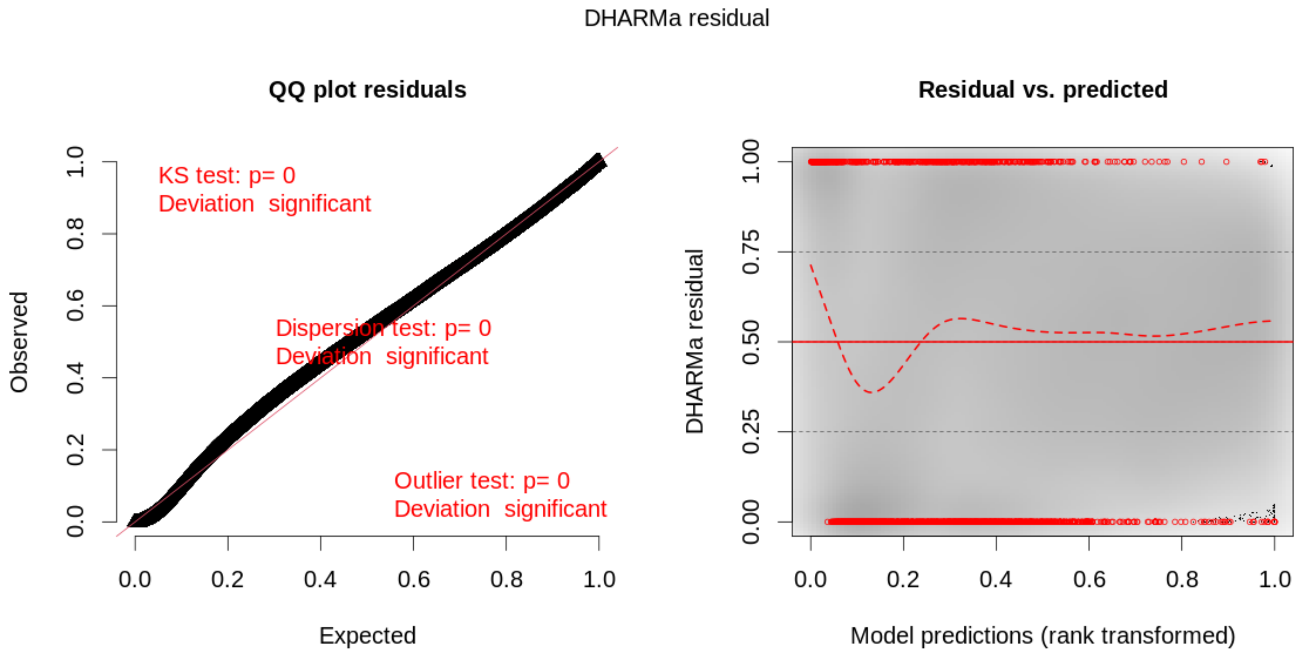

(B) ED attendances

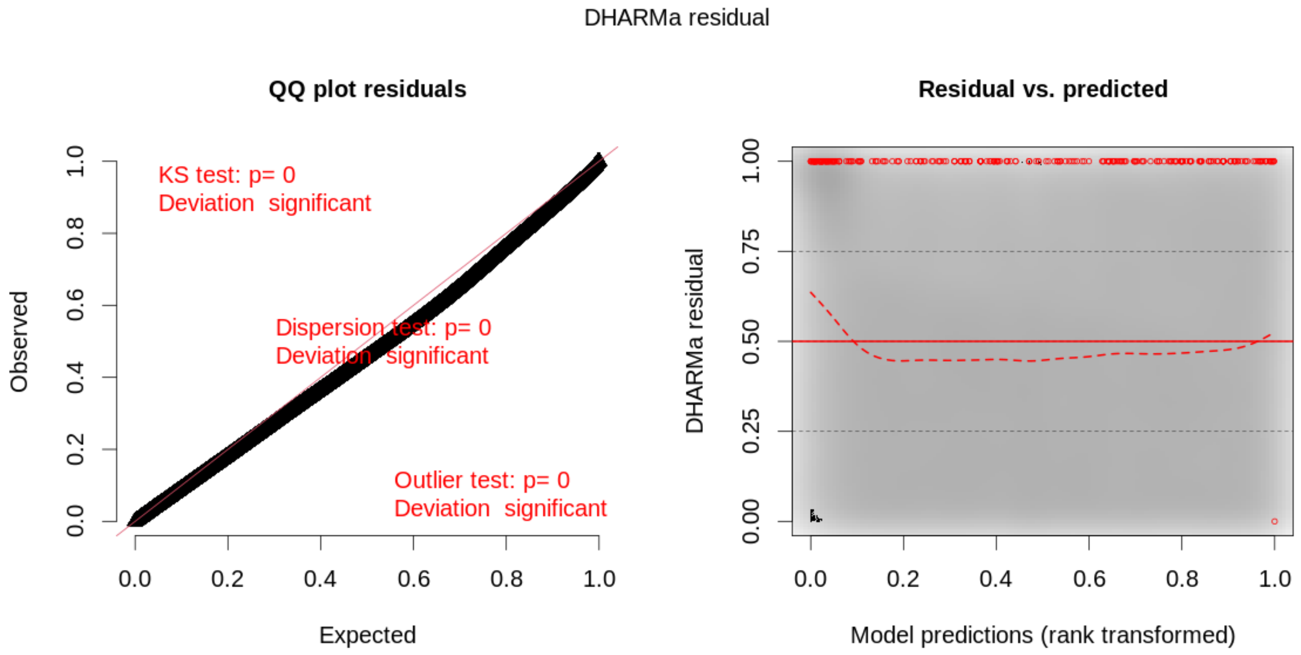

(C)Outpatient attendances

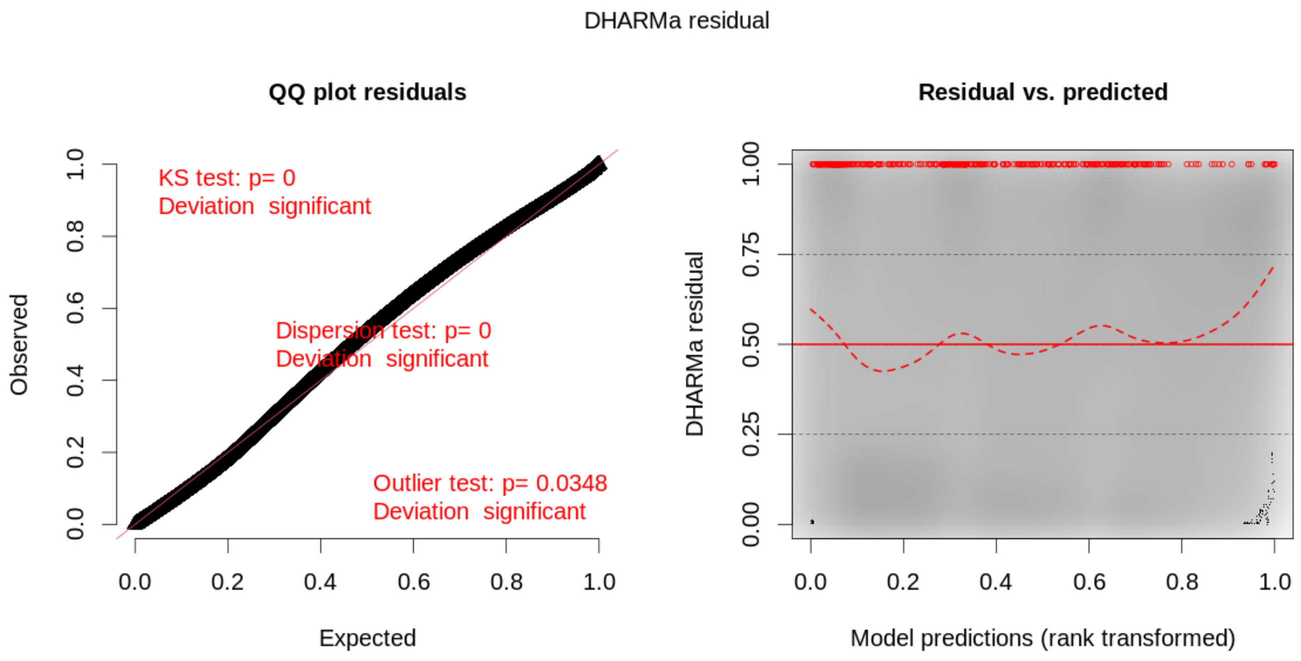

(D) Length of stay in hospital

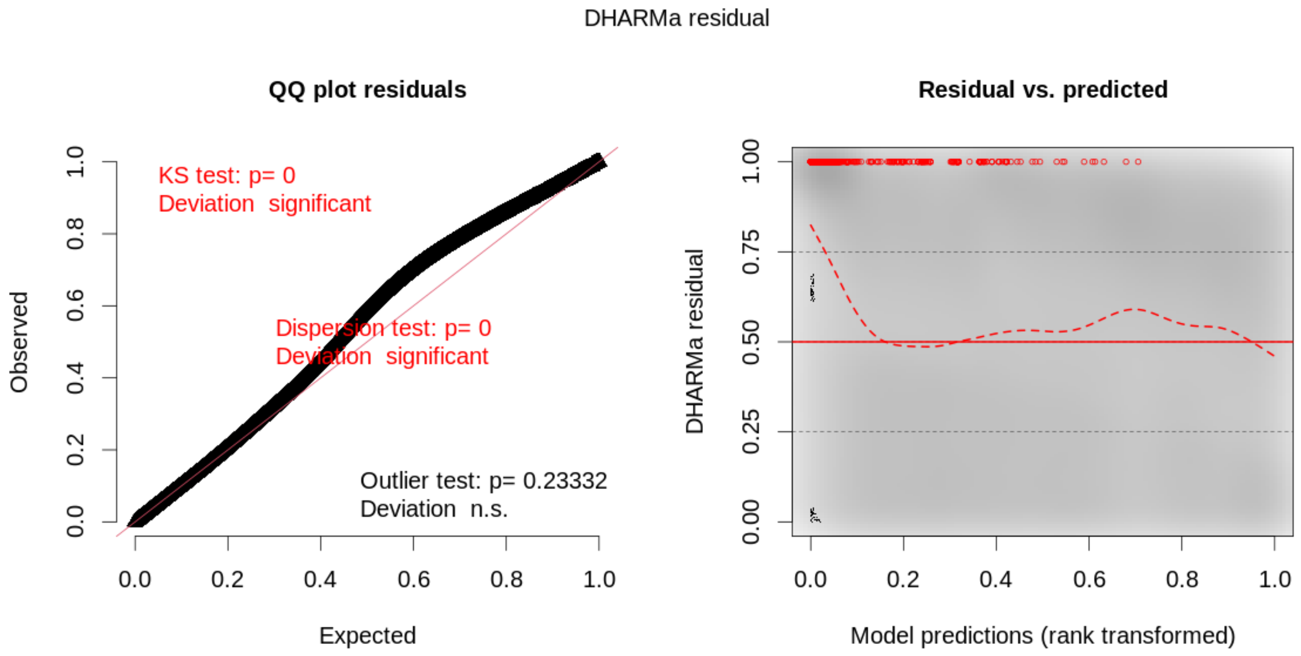

Supplement: Supplementary file 1 [file jcm-12-00872-s001.zip › Figures S1, S2, S3 - diagnostic_plots.pdf]
